# Estimation of Basin-scale turbulence distribution in the North Pacific Ocean using CTD-attached thermistor measurements

**DOI:** 10.1038/s41598-020-80029-2

**Published:** 2021-01-13

**Authors:** Yasutaka Goto, Ichiro Yasuda, Maki Nagasawa, Shinya Kouketsu, Toshiya Nakano

**Affiliations:** 1 Atmosphere and Ocean Research Institute, The University of Tokyo, Kashiwanoha 5-1-5, Kashiwa, Chiba 277-8564 Japan; 2 Research Institute for Global Change, Japan Agency for Marine-Earth Science and Technology, Natsushima 2-15, Yokosuka, Kanagawa 237-0061 Japan; 3 Japan Meteorological Agency, Otemachi 1-3-4, Chiyoda, Tokyo 100-8122 Japan; 4 Present Address: Kushiro Local Meteorological Office, Japan Meteorological Agency, Saiwai-cho 10-3, Kushiro, Hokkaido 085-8586 Japan; 5 Present Address: Nagasaki Local Meteorological Office, Japan Meteorological Agency, Minamiyamate-machi 11-51, Nagasaki, Nagasaki 850-0931 Japan

**Keywords:** Physical oceanography, Physical oceanography

## Abstract

A recently developed technique for microstructure measurement based on a fast-response thermistor mounted on a conductivity-temperature-depth equipment was used on eight cruises to obtain 438 profiles. Thus, the spatial distribution of turbulent dissipation rates across the North Pacific sea floor was illustrated, and was found out to be related to results obtained using tide-induced energy dissipation and density stratification. The observed turbulence distribution was then compared with the dissipation rate based on a high-resolution numerical ocean model with tidal forcing, and discrepancies and similarities between the observed and modelled distributions were described. The turbulence intensity from observation showed that the numerical model was overestimated, and could be refined by comparing it with the observed basin-scale dissipation rate. This new method makes turbulence observations much easier and wider, significantly improving our knowledge regarding ocean mixing.

## Introduction

Ocean turbulence with a vertical diffusivity of *O* (10^–4^ m^2^ s^−1^) is^1^ required to maintain the vertical water-mass distribution in the deep North Pacific. Therefore, quantifying the vertical diffusivity is important to evaluate the meridional overturning circulation and water-mass distribution. A recent modelling study^2^ reproduced an observed Δ^14^C distribution in the deep Pacific using a spatially variable vertical diffusivity distribution, which was constructed on the basis of high-resolution models^3^ for internal gravity waves generated by tides. However, model-based vertical diffusivity has not yet been sufficiently validated via direct microstructure observations owing to their scarcity.

Basin-scale turbulence distributions have been demonstrated^4,5^ using an indirect method that is based on the fine-scale parameterizations^6–10^ of the interior ocean, far from turbulence generation sites (surface and bottom boundary layers, areas close to rough topographies and coastal boundaries, and strong current regions) using fine-scale *O* (10–100 m) density and velocity observations along World Ocean Circulation Experiment-hydrographic sections. Additionally, global horizontal distribution and seasonal variability have been demonstrated using fine-scale density measurements with profiling floats^11,12^, and energy dissipation rate estimations from fine- and micro-scale observations have also been compiled^13^. These studies have revealed that turbulent energy dissipation rates increase over rough bottom topographies, and their spatial variability is related to internal wave energy fields.

While studies based on fine-scale parameterizations have revealed considerable information on turbulence in the deep ocean, the applications of parameterizations are limited to regions where the Garrett–Munk (GM) wave field^14^ is undistorted. Although studies in which distortion was taken into consideration have also been conducted^10^, direct microstructure measurement is still needed to precisely estimate turbulence intensity. However, direct microstructure measurements have been performed only in specific regions, such as ridges and straits, as was the case in the Brazil basin^15^, Hawaiian Ridge^16^, Izu-Ogasawara Ridge^17^, and Kuril Strait^18,19^. Therefore, basin-scale observations using direct microstructure measurements are necessary.

In this study, direct microstructure measurements are demonstrated along eight large-scale hydrographic sections in the North Pacific via the application of the new platform for microstructure observations, which uses conductivity-temperature-depth (CTD) attached thermistors. This system^20–22^ can be used to efficiently evaluate the turbulent energy dissipation rate (*ε*) in the range of 10^–11^–10^–8^ W kg^−1^ with data quality controls considering the effects of the movement of the CTD-frame as well as data adjustment on the attenuation of the temperature gradient spectra. The observation-based turbulence intensity can then be compared with the tide-induced baroclinic energy and the density stratification as well as the energy dissipation rate based on the fine-scale parameterization. Furthermore, it can be quantitatively compared with the dissipation obtained based on the ocean general circulation model (OGCM) so as to clarify the discrepancy between the model and the observations.

## Results

### Turbulence distribution in the North Pacific

**Figure 1:**
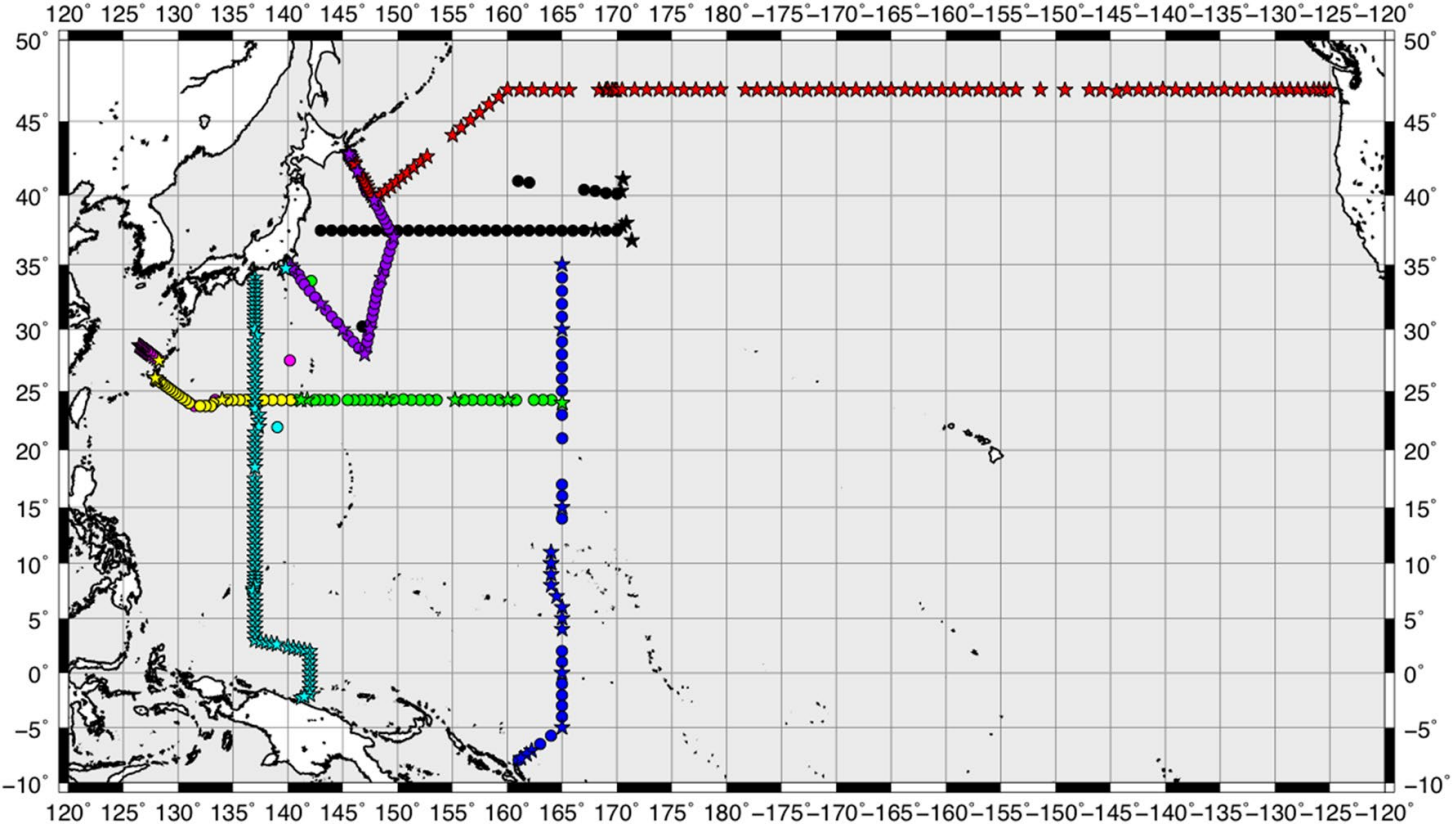
Station locations for 438 CTD-attached fast-response thermistor measurements. The stars denote the full-depth stations, with casts all the way down to the sea floor, and the circles represent the other casts, which extended down to a depth of 2000 m. Stations corresponding to KS16-06 with magenta colour are under KS16-08 (green) and KS16-09 (yellow), and are masked. See Table S1 for cruise codes.

**Figure 2:**
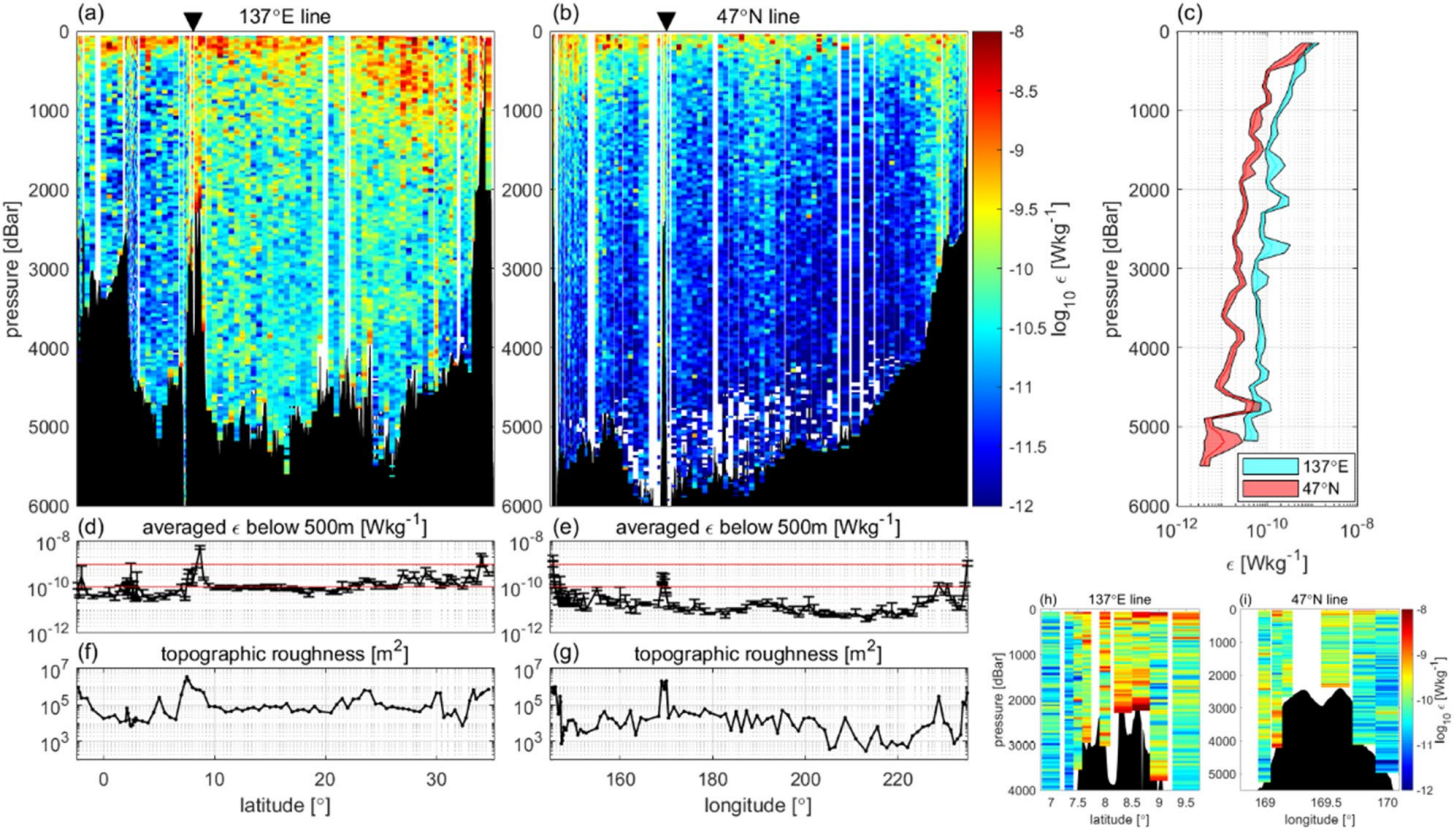
Vertical cross-sections of turbulent energy dissipation rate, *ε*. Along (**a**) 137°E (RF16-06) and (**b**) 47°N (MR-14-04) with triangular marks corresponding to areas close to the Ngulu Atoll and the Emperor Seamount. In (**a**) and (**b**), *ε* is averaged over 50 m after eliminating the data at *W*_sd_ > 0.2* W*–0.06 and *W*_min_. (**c**) Vertical profile of horizontally averaged *ε* for each section. (**d**,**e**) Vertically averaged *ε* below 500 m to exclude surface dissipation, where the red lines denote values at *ε* = 10^–10^ and 10^–9^ W kg^−1^. (**f**,**g**) Topographic roughness at each station, defined as the variance of bathymetric height (m^2^) obtained from ship depth soundings^23^, calculated in 60 km square regions. (**h**,**i**) Enlarged views of the Ngulu Atoll and the Emperor Seamount.

Two basin-scale, vertical cross-sections of *ε* from fast-response thermistor measurements along 137°E and 47°N (Fig. 1 for station locations) are shown in Fig. 2a,b, respectively, with the vertical profiles of *ε* averaged in each section (Fig. 2c). The overall features of *ε* were as follows: (1) vertically, *ε* was found to be generally large at depths from the surface to the pycnocline. (2) The 137°E-mean *ε* at each depth was three times that in 47°N (Fig. 2a–c). (3) Relatively large *ε* values were observed over rough bottom topographies, such as the Ngulu Atoll at 8°N 137°E, the Emperor Seamount at 47°N 170°E, and the slope edge at 47°N 50°W. Quantitatively, *ε* averaged from a depth of 500 m to the bottom (Fig. 2d–e) corresponded to the topographic roughness represented by the variance of the bottom depth (Fig. 2f–g). These features obtained using the present CTD-attached method are similar to those reported in previous studies^4,5,11–13^.

**Figure 3:**
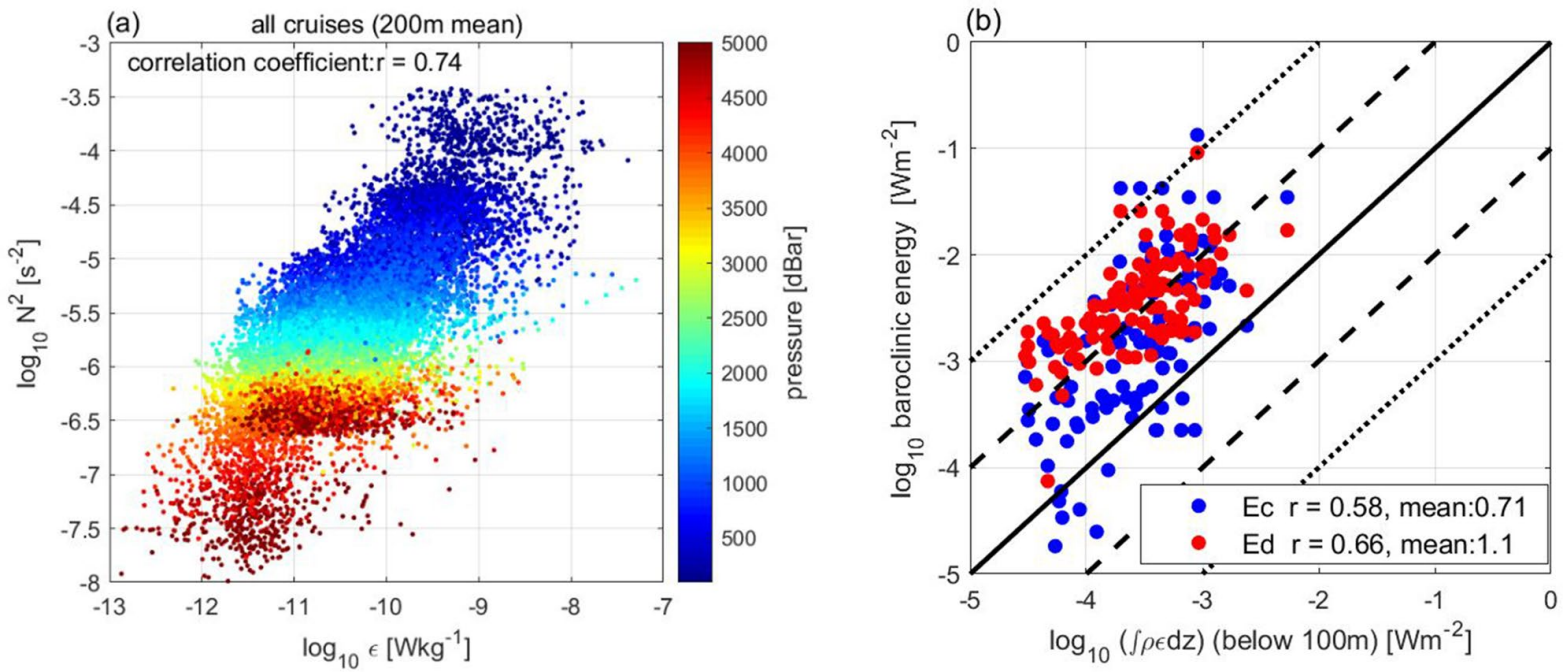
Relationship between *ε* and *N*^2^ and baroclinic tide energy. (**a**) Relationship between the observed energy dissipation, *ε*, and the squared buoyancy frequency, *N*^2^. Colours denote pressure. (**b**) Relationship between depth-integrated observed energy dissipation, ∫*ρε*d*z*, and model-based depth-integrated baroclinic energy conversion rate, *Ec* (blue dots), representing the generation of internal waves by tidal forcing, and the relationship between ∫*ρε*d*z* and depth-integrated tide-induced energy dissipation, *Ed* (red dots), both correspond to MR-14-04 and RF16-06. X-axis: depth-integrated *ρε* from a 100-m depth to the bottom, excluding the surface 100-m layer to avoid the influence of wind. Y-axis: *Ec* and *Ed* are estimated in the model^2^ based on the 3-D tide-driven model^3^. *Ec* and *Ed* simulated with the resolution (1/15°) model are multiplied by 1.5 as in the model^2^. “r” in the legend represents the correlation coefficient in logarithmic scale and “mean” is the average of log(Y/X).

Further analysis of feature (1) showed that the observed *ε* correlated well with the strength of stratification (Fig. 3a), i.e. the squared buoyancy frequency, *N*^2^ (s^−2^) (= *gα*(∂*T*/∂*z* + *γ*) − *gβ*∂*s*/∂*z*, where *g* represents the gravitational acceleration (m s^−2^), *T* represents temperature (°C), *s* represents salinity (psu), *α* represents the coefficient of thermal expansion (°C^−1^), *β* represents the coefficient of saline contraction (psu^−1^), and *γ* represents the adiabatic lapse scale (°C m^−1^), with the high correlation coefficient, *r* = 0.74 (see Table S2 in the “Supplementary Information” for statistical data).

For features (2) and (3), the horizontal distribution of the depth-integrated observed dissipation, ∫*ρε*d*z*, depended on the energy distribution of the internal waves driven by tidal forcing (Fig. 3b). Depth-integrated observed energy dissipation, ∫*ρε*d*z*, from the full-depth casts was found to be correlated (*r* = 0.58) with the model-based horizontal energy distribution of the internal wave generation *Ec*(*x*,*y*) (see “Methods”)*.* It was also found to be correlated (*r* = 0.66) with the horizontal distribution of the model-based energy dissipation, *Ed*(*x*,*y*), estimated using tide-forced internal waves. These relationships indicate that the depth-integrated observed dissipation, ∫*ρε*d*z*, is regulated by the energy distribution of the tide-induced internal waves. The relationship between the observed *ε* and topographic roughness was explained by the tide-generating internal wave energy distribution. The higher internal wave activity along 137°E seemed to induce a relatively higher *ε* averaged over the sections than along 47°N (Fig. 2c).

Close to the sea floor, *ε* values were not high. However, bottom-intensified mixing has been demonstrated in previous studies^24^. One possible reason for the low *ε* values could be the lack of data corresponding to the area close to the sea floor. Since the frame falls slowly within 100 m above the sea floor, most data were eliminated by the data rejection criterion of *W*_sd_ > 0.2*W–*0.06 (see “Methods”); thus, the data became sparse. These findings can be attributed to the absence of the bottom-intensified dissipation structure, which was modelled as maximum dissipation at the bottom and decayed exponentially with height^25^. Further analysis of the data quality and the improvement of the analysis method are required to quantify the turbulence, 100 m above the sea floor. Another possible reason is that roughness is not sufficient to generate strong turbulence in many stations along 137°E and 47°N, except in areas with very rough topography, such as the Ngulu Atoll and the Emperor Seamount (Fig. 2h,i).

### Comparison with fine-scale parameterizations

**Figure 4:**
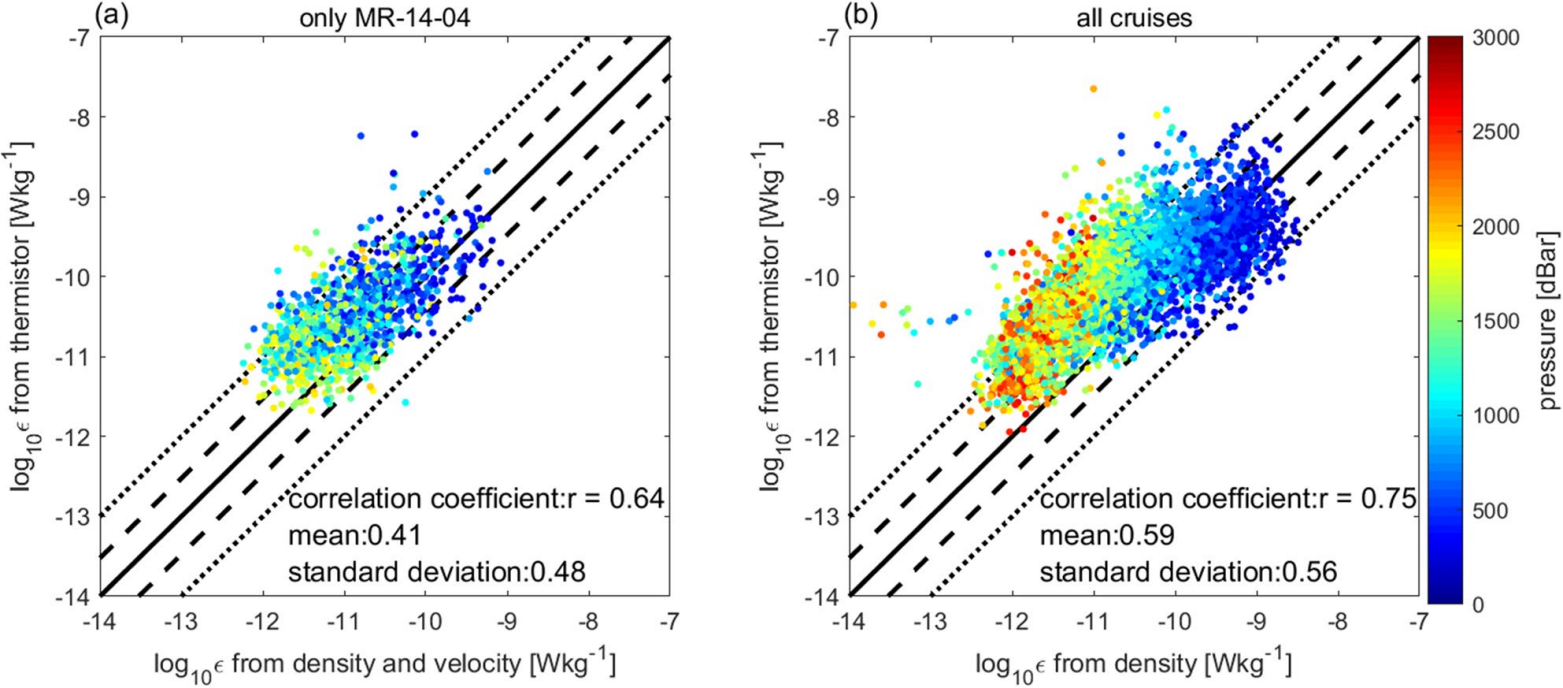
Comparison of *ε* with fine-scale parameterizations. (**a**) *ε* based on fine-scale velocity and density using Lowered Acoustic Doppler Current Profilers (LADCP) and CTD^10^. (**b**) *ε* based on only fine-scale density^8^. Data are averaged in 320-m segments. The surface data that included the upper 100-m layer are not used. In (**a**), data with pressure > 2000 dbar are excluded because the accuracy of the measurement of LADCP is low in the deep ocean. In (**b**), data with *N*^2^ < 10^–6^ s^−2^ are excluded since the estimation of the vertical gradient of density is difficult in the low stratified regions. “mean” and “standard deviation” in the legend represent the average and the standard deviation of log(Y/X), respectively.

Most data were obtained in the interior ocean, where fine-scale parameterizations^6–10^ are applicable. Consistency between the present method and the previous fine-scale parameterization method can support the validity of the new CTD-attached thermistor method. Along the 47°N section, where velocity data are available, we calculated *ε*_FINE_ using a new method^10^ based on fine-scale velocity and density profiles, while considering the spectral distortion depending on the ratio of fine-scale kinetic to potential energy (*R*_ω)_. The calculated *ε*_FINE_ values were found to be correlated with the *ε* values obtained using the CTD-attached method (*r* = 0.64), and the average of log(*ε*/*ε*_FINE_) was within a factor of three (Fig. 4). For the data corresponding to other sections, even the method involving the use of only density data^8^ resulted in high correlation (*r* = 0.75), without the abnormal overestimation of *ε*.

### Comparison with *ε*-field used in ocean circulation model

The observed *ε* distribution was compared with *ε*_TideNF_ (see “Methods”) used in the ocean circulation model^2^, which reproduced the Δ^14^C distribution in the deep Pacific (TideNF model). The vertical *ε*_TideNF_ distribution close to the tide generation sites, *ε*_NEAR_, was assumed to exponentially decay above the sea floor with a decaying scale, *h* (= 500 m), and the vertically integrated dissipation at each location was set to the local dissipation rate, *q* (= 1/3), of the baroclinic energy conversion rate *Ec*(*x*,*y*) from the barotropic tides. Parameters *h* and *q* were based on previous observational and numerical studies^24,25^ and *Ec*(*x*,*y*)^3^. The vertical *ε*_TideNF_ distribution far away from the tide generation sites, *ε*_FAR_, was assumed to be vertically uniform^2^ for simplicity. The background *ε*_TideNF_ distribution, *ε*_BACK_, was assumed to have the vertically uniform diffusivity^2^, *K*_BACK_ (= 10^–5^ m^2^ s^−1^). Please refer to the “Methods” section for the details on *ε*_TideNF_.

**Figure 5:**
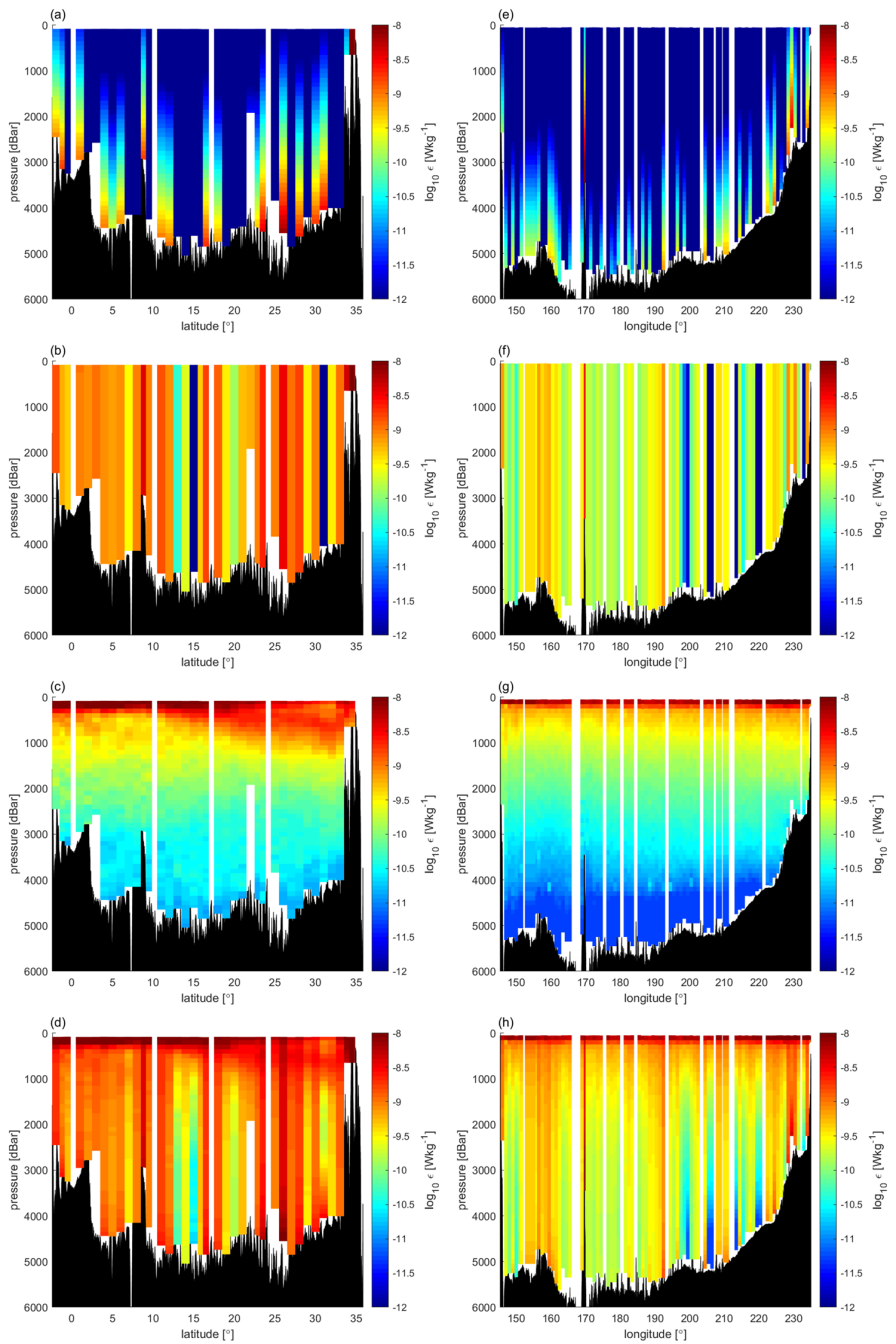
*ε*_TideNF_ along 137°E (left) and 47°N (right). (**a**,**e**) Near-field, *ε*_NEAR_, (**b**,**f**) Far-field, *ε*_FAR_, (**c**,**g**) Background, *ε*_BACK_, (**d**,**h**) *ε*_TideNF_ = *ε*_NEAR_ + *ε*_FAR_ + *ε*_BACK_. The squared buoyancy frequency is observation-based, and the mixing efficiency, Γ, is 0.2.

Notably, *ε*_TideNF_ was much higher (over ten times) than the observed *ε* (Fig. 5), while the correlation coefficient between them for depths below 500 m based on data from eight cruises was *r* = 0.55, suggesting that spatial variability has some similarity with the observations. The relatively high *ε*_NEAR_ over the rough topographies (such as the Ngulu Atoll at 8°N 137°E, the Emperor Seamount at 47°N 170°E, and the slope edge at 47°N 50°W) were estimated in the model; it was observed that they contributed to the representation of the observational features of the spatial changes in ε (Figs. 2a,b and 5a,e). Considering that the constant background diffusivity was used in the model, it was observed that *ε*_BACK_ (= *K*_*ρ*_*N*^2^Γ^−1^ = 10^−5^*N*^2^ × 0.2^–1^) depends on the strength of stratification, *N*^2^. Thus, *ε*_BACK_ contributed to the representation of the dependency of stratification (Figs. 2a,b and 5c,g), which was also detected in ε (the correlation coefficient between log(*ε*) and log(*N*^2^) was 0.74; Fig. 3a). Further, *ε*_FAR_, which was assumed to be vertically uniform in the model^2^, showed the greatest deviation from the observed *ε*, and had an order of magnitude that was larger than that of the observed *ε*, even far from the rough bottom topography (Figs. 2a,b and 5b,f).

**Figure 6:**
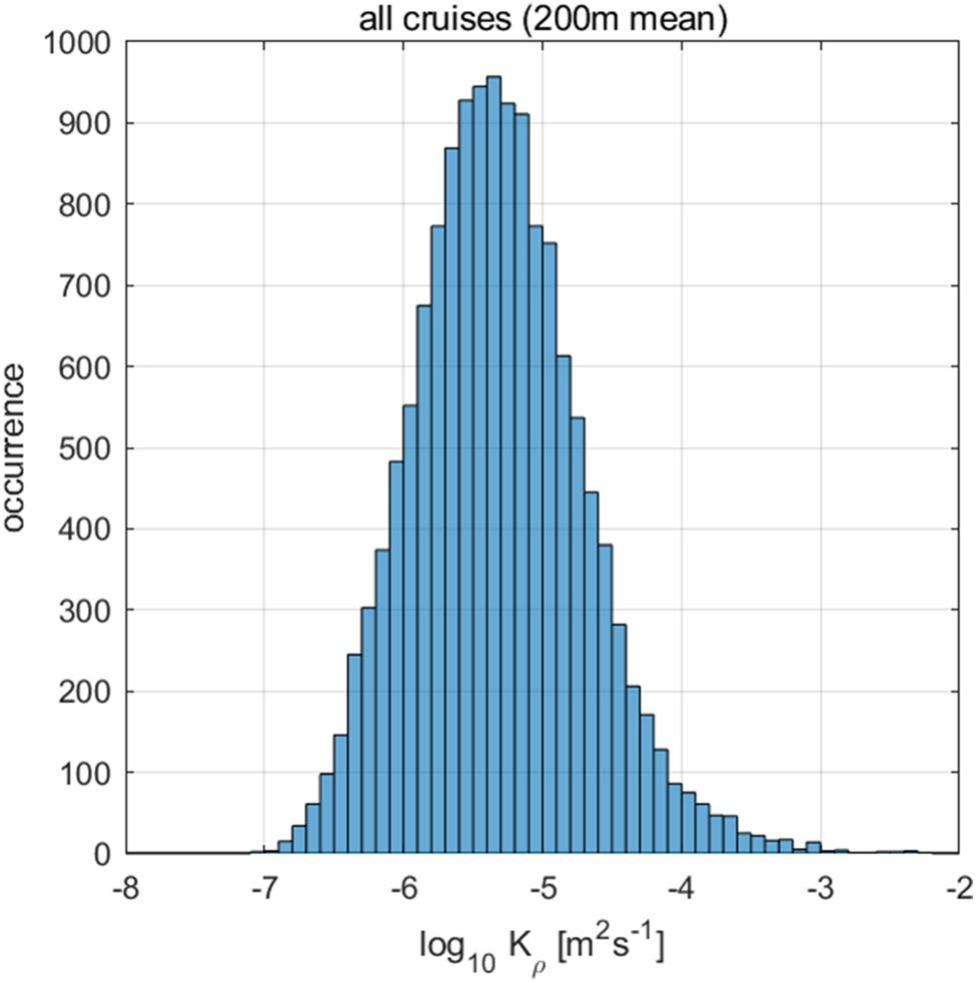
Occurrence of diapycnal diffusivity *K*_*ρ*_ (= 0.2*ε*/*N*^2^). The vertical axis denotes the data quantity. Data averaged at 200 m after data screening are displayed.

**Figure 7:**
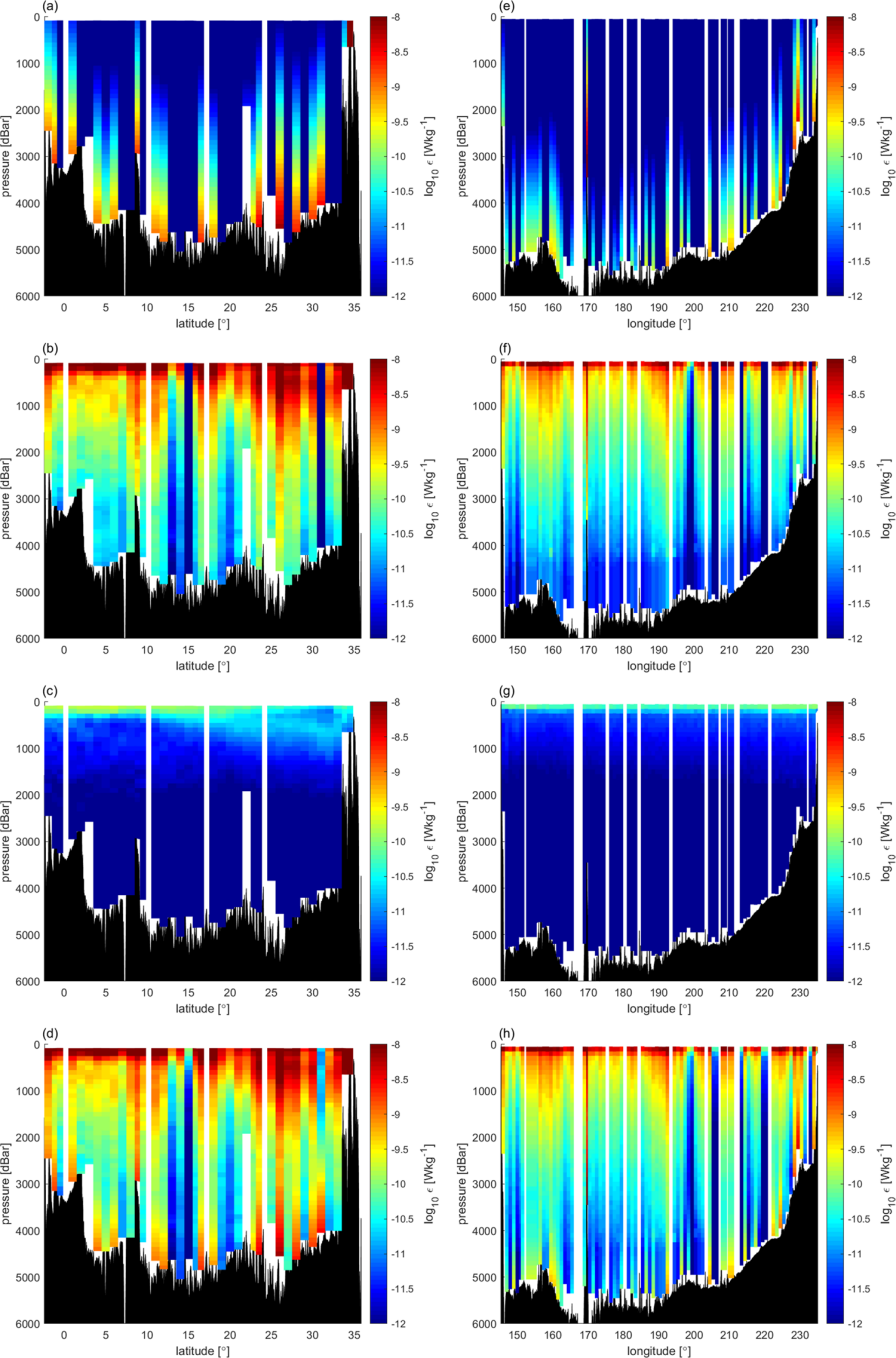
Modified *ε*_MOD_ distribution adjusted to the observed *ε*. (**a**,**e**) Near-field *ε*_NEAR_, (**b**,**f**) Far-field *ε*_FAR_, (**c**,**g**) Background *ε*_BACK_, (**d**,**h**) *ε*_MOD_ = *ε*_NEAR_ + *ε*_FAR_ + *ε*_BACK_.

**Figure 8:**
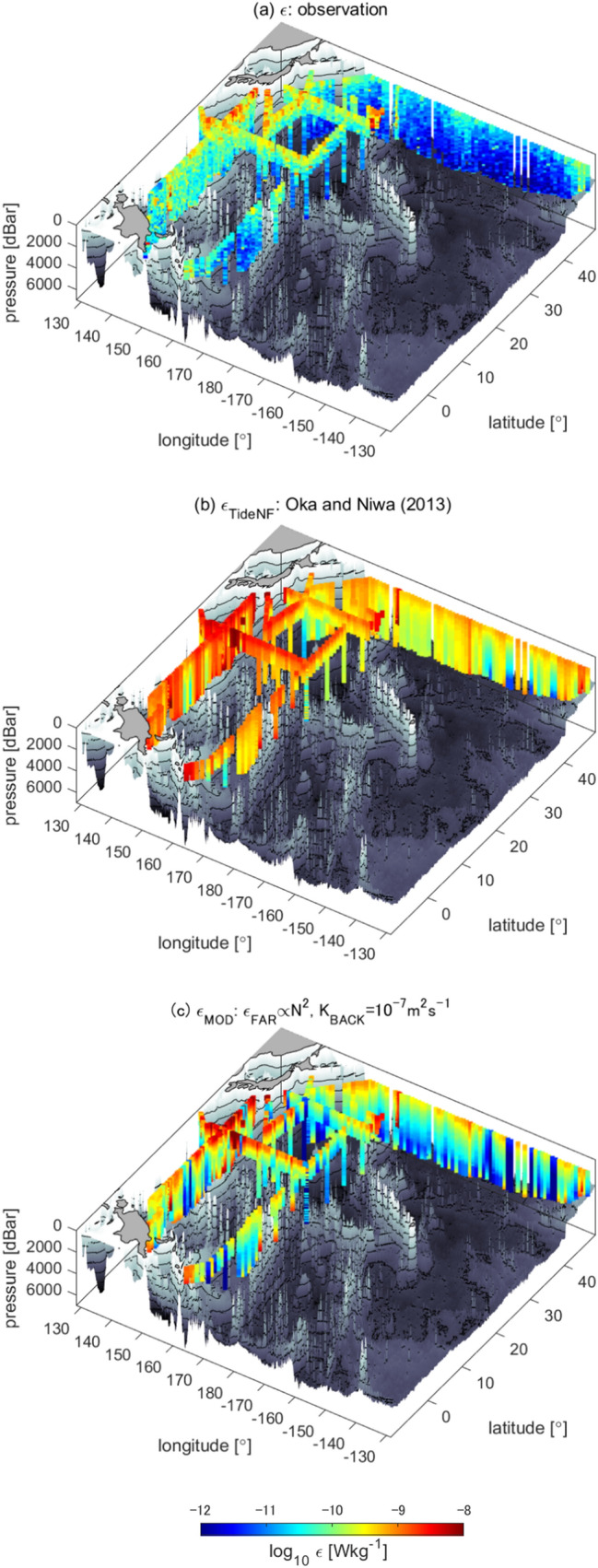
Comparison of 3-D *ε* distributions. Comparison of *ε* (**a**) Based on the CTD-attached thermistor observations, (**b**) based on the OGCM^2^, which reproduced the radio-carbon isotope ratio in the Pacific, and (**c**) based on the modified model, within which the far-field *ε* is proportional to the squared buoyancy frequency, *N*^2^, and the background value of *ε* is based on the observed minimum diapycnal diffusivity, *K*_*ρ*_ = 10^–7^m^2^ s^−1^, to make the model distribution closer to the observations.

In the model^2^, while the near-field diffusivity was set following the parameterization^25^ and considering the observations^24^, the background and far-field diffusivity were not based on direct observations, but were set so that the predicted tracer field follows the observations. Thus, we introduced another parameterization to modify *ε*_TideNF_, referred to as *ε*_MOD_. The vertical structure, *F*_FAR_(*z*), of *ε*_FAR_ (Eq. 2 in “Methods”) was modified to be proportional to the squared buoyancy frequency, *N*^2^, as *F*_FAR_(*z*)$=({N}^{2}\text{/}\overline{{{\text{N}}}^{2}}\text{)}{{\text{H}}}^{-1}$, where the overbar denotes the vertical mean and *H* represents the sea floor depth^26^. This form is consistent with the observational fact that *ε* is correlated with *N*^2^ (Fig. 3a). Background diffusivity was then changed to *K*_BACK_ = 10^–7^ m^2^ s^−1^ given that the minimum value of the observations was 10^–7^ m^2^ s^−1^ (Fig. 6). After revision, *ε*_MOD_ became closer to the observed *ε* within a factor of three and with a relatively high correlation coefficient (Fig. 7; *r* = 0.50 between Fig. 8a,c), whereas large discrepancies were observed between *ε*_TideNF_ and the observed *ε* (Fig. 8a,b). In the revised *ε*_MOD_, *ε*_FAR_ was found to be the main contributor to the major part of the ocean, except at depths close to the sea floor, where *ε*_NEAR_ showed dominance, with *ε*_BACK_ being very small (Fig. 7b,f). This modification implies that the baroclinic energy generated at the bottom propagates more widely with the longer damping time scale compared to the TideNF model.

## Discussion

There were uncertainties in both the observation- and model-based estimates. One reason for these uncertainties was the variable response time of the FP07 thermistors. In this study, to capture micro-scale turbulence, all temperature gradient spectra were corrected and multiplied by the double-pole low-pass filter function [1 + (2*πfτ*)^2^]^2^ with the time constant, *τ* = 3 ms, to compensate for the attenuation of the frequency spectra owing to the insufficient response speed. The time constants of the individual FP07 sensors could be varied between 1.9 and 5.6 ms for the double-pole functions at 1/4 attenuation (“Methods”). Another uncertainty arose in the estimation of *ε* in the weak turbulence range of *ε* < 10^–10^ W kg^−1^. This is because such weak turbulence could not be evaluated based on micro-scale shear observations, which were used for the validation of the FP07 observations^20^. Thus, *ε* in this range varied between 0 and 10^–10^ W kg^−1^.

The uncertainties associated with the response time and weak turbulence range were evaluated by comparing the geometric means of the ratios of *ε* (Table 1), which could be varied by changing *τ* and/or *ε* in the range *ε* < 10^–10^ W kg^−1^. When *τ* was changed, and keeping *ε* < 10^–10^ W kg^−1^, i.e. *ε*_BACK_ (the middle column), the ratio varied from 0.398 to 1.49, and the uncertainty factor was approximately four. In contrast, when the background *ε* was changed and *τ* was kept constant (*τ* = 1.9), and the uncertainty factor was approximately eight (the ratio varied between 0.398 and 3.13). Even though all the differences among these cases were within a factor of ten these results support the importance of time constant estimates for each probe as well as precise measurements of weak turbulence below 10^–10^ W kg^−1^ in deep ocean.

**Table 1:** Uncertainties in *ε* based on observations.

	*ε* < 10^–10^ W kg^−1^ is unchanged (original)	*ε* < 10^–10^ W kg^−1^ is *ε*_BACK_ (minimum)	*ε* < 10^–10^ W kg^−1^ is 10^–10^ (maximum)
*τ* = 1.9 (ms)	0.742 (0.738–0.745)	0.398 (0.392–0.404)	3.13 (3.06–3.19)
*τ* = 3.0 (ms)	1	0.572 (0.563–0.582)	3.70 (3.63–3.77)
*τ* = 5.6 (ms)	2.23 (2.21–2.25)	1.49 (1.46–1.53)	6.18 (6.10–6.26)

The geometric means of the ratios between the modified *ε* (changing *τ* and *ε* in *ε* < 10^–10^ W kg^−1^) and the original *ε* (the case of the middle row, the left column), with 95% confidence intervals of the bootstrap method are in the parentheses. The 200 m-mean data set of the *ε* values corresponding to all observation points of the eight cruises were used. The three values correspond to the uncertainty of the individual thermistors whose time constant based on spectrum double-pole correction ranges between 1.9 and 5.6 ms. Another source of uncertainty is the weak turbulence region, *ε* < 10^–10^ W kg^−1^, where *ε* is set to the background value (the middle columns), *ε*_BACK_, or fixed at 10^–10^ (the right column). *ε*_BACK_ is based on the observed minimum diapycnal diffusivity, i.e. *ε*_BACK_ = *K*_*ρ*_·*N*^2^·Γ^−1^ = 10^–7^·*N*^2^·0.2^–1^.

It was also necessary to consider the temporal variability in turbulence intensity. The observation at each station in this regard was performed only once. Actually, temporal variability with semi-diurnal and diurnal tidal cycles exists. For example, in strong turbulence regions such as the Kuril Strait, the temporal variability was larger than two orders of magnitude, and was related to the tidal cycle^18^. It was expected that the distribution of *ε* in our observations will likely vary if the observations were made at the same stations. Thus, to obtain representative *ε* values in such strong turbulence regions, further observations should be performed to estimate the temporal average.

The local dissipation ratio, *q* (= 1/3), of the generated internal tide energy, *Ec*(*x*,*y*), and decay scale, *h* (= 500 m), in near-field *ε*_NEAR_ have been proposed in previous numerical and observational studies^24,25^. The decay scale, *h*, may not be constant, but might depend on the amplitude of tidal flow and the horizontal wavenumber of the bottom topography^27^. The data corresponding to this study were insufficient to determine *h* because a large portion of the CTD casts could only descend to a depth of approximately 2000 m, failing to reach the sea floor. Furthermore, the dissipation rate *ε*, from most of the full-depth casts decreased toward the bottom and did not exhibit the exponential decay structure from the bottom. The bottom-enhanced structure was observed only in the Ngulu Atoll at 8°N 137°E and the Emperor Seamount at 47°N 170°E, both of which are strong internal tide generation sites with high baroclinic energy conversion from barotropic tides. To consider the spatial differences in *h*, it was necessary to accumulate bottom-reaching *ε* data. Additionally, in this study, parameter *q* was also constant; possibly dependent on the latitude because diurnal internal waves cannot propagate at high latitudes with a possibility of local dissipation intensifying^28,29^. To consider the latitudinal dependence of *q*, meridional observations at the sea floor covering high latitudes are required.

For far-field dissipation, *ε*_FAR_, the vertical structure proportional to *N*^2^ in *ε*_MOD_ was more suitable for the reproduction of the observational distribution than the simple vertically uniform *ε*_FAR_ in *ε*_TideNF_. This dependency on *N*^2^ is consistent with the theory^6^, where *ε* ∝ *N*^2^*E*^2^ (*E* represents the fine-scale energy density) in the GM internal wave field^14^. Additionally, the theory^6^ served as the basis for the existing fine-scale parameterizations^7–10^. However, further research is required to explain how this *N*^2^-dependence is established from tide-induced internal wave fields.

It is worth noting that the model-based energy dissipation *Ed*(*x*,*y*), and baroclinic energy conversion *Ec*(*x*,*y*), were much greater than the depth-integrated observed ∫*ρε*d*z* regardless of the high correlation coefficients. *Ec* (*Ed*) was 5 (13) times greater than $\int_{{{\text{bottom}}}}^{{100\;{\text{m}}}} {\rho \varepsilon {\text{dz}}}$ (Fig. 3b). This magnitude of difference has also been reported between directly measured *ε* and model-based forcing and dissipation estimates^13^, suggesting that dissipation takes place in locations other than the observed sites, or close to the bottom or the surface, where the present observations did not fully cover. However, this will be a subject of future studies owing to the uncertainties in both the observations and models.

The background diffusion obtained in model^2^ and those obtained in other modelling studies^29,30^ *K*_BACK_ = 10^–5^ m^2^ s^−1^, were two orders of magnitude larger than the minimum value obtained in this study, and the total background energy dissipation accounted for more than 20% of the total dissipation in the upper 1000 m. *K*_BACK_ was reduced to the observed minimum in this study. *K*_BACK_ could increase if the dissipation by the near-inertial internal waves forced by winds^31^ or internal lee waves due to current-topography interactions^32^ were included in *ε*_BACK_. Although the dissipation related to *N*^2^ was included in a component of the tide-generated dissipation *ε*_FAR_, which constituted a major part of the internal tide-induced energy dissipation in this study, it might have been appropriate to include it in *ε*_BACK_ if the wind and internal lee waves have a large contribution to the dissipation related to *N*^2^.

In this study, direct microstructure observations were conducted in the deep North Pacific using fast-response thermistors attached to CTD frames. After careful data quality control, through which data resulting from the movement of the frames was eliminated, the basin-scale distribution of turbulence energy dissipation rate *ε*, was revealed. High *ε* values were observed over the rough topography close to seamounts and ridges, where reportedly, internal tides are generated. The *ε* values from the micro-temperature were found to be comparable with that of the previous fine-scale parameterization estimation in the interior ocean within a factor of three. Additionally, they also showed correlation with the internal tide energy dissipation and the squared buoyancy frequency, *N*^2^. However, these *ε* values were 1/10 times smaller than those obtained using the previous OGCM, which reproduced the deep Pacific water-masses fields. To adjust *ε*_TideNF_ to the observed *ε*, we proposed another parameterization. By adjusting the vertical structure that is far from the internal tide generation sites (far-field) to be proportional to *N*^2^, and by reducing the background constant diapycnal diffusivity to 10^–7^ m^2^ s^−1^, the difference was modified to be within a factor of three. This indicates that widespread observations using the CTD-attached thermistors with higher spatial and temporal resolutions can contribute to a more realistic representation of diapycnal diffusivity distribution in OGCMs in the near future.

## Methods

### Observational data

A total of 438 vertical profiles of micro-temperature data were obtained during the cruises of the R/V Keifu Maru, R/V Ryofu Maru, R/V Mirai, and R/V Hakuho Maru vessels in the North Pacific (see Table S1 in the “Supplementary Information”). Full-depth observation of the sea floor was performed at all the stations along the 47°N and 137°E sections. In the other sections, measurements were performed at several stations down to a depth of 2000 m, with some being full-depth observations. For each location, a one-time measurement was performed. The station locations are shown in Fig. 1.

The micro-temperature profilers, Micro Rider 6000 and AFP07, both manufactured by Rockland Scientific Inc. (Canada), were installed on CTD frames of the cruises. Micro Rider 6000 was installed in the MR-14-04 cruise and AFP07 was installed in the remaining cruises. To avoid measurement of artificial turbulence caused by the frames, the two fast-response thermistors (Fastip Probe model 07; henceforth FP07) were attached close to the bottom of the frames (Fig. 1 of the study^21^). Probes whose spectra fit the universal spectrum better was used in this analysis.

### Estimation of the energy dissipation rate, *ε*

Turbulent energy dissipation rates (*ε*) were estimated using the relationship, *ε* = (2π)^4^*k*_B_^4^*νκ*^2^, where *ν* represents the kinematic viscosity (m^2^ s^−1^), *κ* represents the molecular thermal diffusivity (m^2^ s^−1^), and *k*_B_ represents the Batchelor wavenumber of the temperature spectrum (cpm). We estimated *k*_B_ by fitting the universal spectrum to the observed temperature gradient spectrum^33,34^. The data processing was the same as in a previous study^21^, based on the maximum likelihood estimation method^35^. Each spectrum was determined from a profile segment within 1 s and corrected using the double-pole low-pass filter function^36^. Considering that each thermistor was not calibrated, the time constant, which represents the effect of smoothing the microstructures due to relatively slow sensor response, was fixed at 3 ms^20^.

Time constant dependence of the sensor fall rates has been demonstrated in some previous studies^36,37^. The faster the sensor fall rate, the smaller the time constant becomes; thus, the required correction is also smaller. However, making corrections considering this characteristic could result in underestimation in areas with strong turbulence^21^. When the sensor falls with a higher speed, higher frequencies are necessary to determine the Kraichnan spectrum. These higher frequencies could be significantly attenuated, making it difficult to attain full correction, regardless of the use of double- or single-pole functions. Consequently, the smaller correction associated with the higher speed results in underestimation. Accordingly, the dependence of the time constant on the sensor speed was not considered in this study.

To estimate *k*_B_, we only used the spectrum in the lower frequency domain avoiding using the high frequencies dominated by electrical noise, which was determined by comparing the noise spectrum obtained with dummy probes in our laboratory. The form of the fitted universal spectrum was the one^34^ with the fixed universal constant, *q*_K_ = 5.26^38,39^. After the fitting, automatic rejection criteria^40^ based on the shape of the observed spectrum were applied on each spectrum to eliminate poorly fitted data.

### Data screening

Given that the CTD-attached fast-response thermistor is a new platform for the measurement of turbulence, a specific method for quality control was necessary. At times, not-free-fall measurements generate artificial turbulence due to the CTD-frame or probes themselves, causing the overestimation of ε as discussed in a previous study^21^. Such overestimation is frequently observed under the small descending rate [*W* (ms^−1^)] and/or the large standard deviation of *W* [*W*_sd_ (ms^−1^)], when data from the R/V Hakuho Maru and R/V Shinsei Maru are used. By eliminating the data satisfying *W*_sd_ > 0.2*W *− 0.06, the CTD-attached thermistor could estimate the energy and thermal dissipation rate in a manner comparable to that of a free-fall thermistor^21^. However, conditions generating artificial turbulence depend on the mode of operation, which might be different from one research vessel to another^22^. Data from the three other ships, the R/V Ryofu Maru, R/V Keifu Maru, and R/V Mirai, in addition to those from the R/V Hakuho Maru were used in this study. Therefore, it was expected that the turbulence data quality would be maintained. Here, we updated the condition for the elimination of the outliers by re-examining the data from the R/V Ryofu Maru (Supplementary Fig. S1).

Before data screening (Fig. S1b), relatively large *ε* values (> 10^–8^ W kg^−1^) were observed in the entire depth range at latitudes 2–3°N, 13°N, 17–19°N, and 24–30°N, corresponding to the large *W*_sd_ (Fig. S1a), which could be caused by ship rolling or pitching due to large surface waves owing to rough sea states. These overestimated *ε* values were efficiently removed by the rejection criteria, *W*_sd_ > 0.2* W *− 0.06^21^ (Fig. S1c). However, large *ε* patches remained at the intermediate-to-deep levels as well as in the casts with large *W*_sd_.

When the vertical profiles of the *ε* values based on the CTD-attached method as well as the fall rate, *W*, and *W*_sd_ were carefully examined, the spikes of the *ε* values were eliminated based on the rejection criteria^21,40^ (the red dots in Fig. S2). However, in areas with small *W* and *W*_sd_ values, unnaturally large *ε* values, above 10^–8^ (W kg^−1^) remained. Reportedly, decelerating *W* can cause artificial turbulence with a higher downward momentum, and such turbulence is considered to keep up with the sensors at around the local minimum of the fall rate *W*_min_ (also in Fig. 7 in the paper^21^). Overestimated data at *W*_min_ were not necessarily removed by the rejection criterion *W*_sd_ > 0.2*W *− 0.06 because *W*_sd_ can also be very small in some cases. By eliminating data at *W*_min_ in addition to that in the range *W*_sd_ > 0.2*W *− 0.06, almost all the spurious *ε* values were removed (Fig. 2d). These combined rejection criteria of *W*_sd_ > 0.2*W *− 0.06 and *W*_min_ were used in this study.

### Dependence of *ε* on individual thermistors

Temperature spectra obtained from thermistors are attenuated at high frequencies; thus, they need to be corrected. The form of the correction functions were single- or double-pole low-pass filters^36,41^ with the time constant, *τ*, which is different for individual probes of the same type due to the differences in glass coatings^42^. Since *ε* varies as *τ* changes, it was necessary to consider the range of *τ* within which the probe was not calibrated. In this subsection, the uncertainty of *τ* was found to be between 1.9 and 5.6 ms in the double-pole low-pass filter function based on the following analysis. The *ε* from seven FP07 thermistors (*ε*_T_) were compared with the *ε* values measured simultaneously using shear probes (*ε*_S_). The free-fall vertical microstructure profiler (VMP2000) manufactured by Rockland Scientific Inc. was employed in the four cruises (Table S3). Data processing was based on the results of a previous study^20^.

It is worth noting that *ε*_T_ is consistent with *ε*_S_ within a factor of three for all the thermistors examined in this study. Although the dependence of the *ε*_T_ estimate on individual thermistors is evident as shown in Fig. S3, some thermistors (serial number (S/N) 886, 1024, and 1025) showed that *ε*_T_ is larger than *ε*_S_ in the entire range of 10^–10^ < *ε*_S_ < 10^–7^ W kg^−1^, whereas other thermistors (S/N 271 and 415) showed smaller values, even after all the temperature gradient spectra were corrected using the same single-pole^41^ (SP: [1 + (2*πfτ*)^2^]) and double-pole^36^ (DP: [1 + (2*πfτ*)^2^]^2^) correction functions with *τ* equal to 7 and 3 ms, respectively (Fig. S3a,b). This scatter was likely caused by the time constant, *τ*, owing to differences in the glass coatings^42^. The degree of the scatter depends on turbulence intensity, *ε*_S_, and the difference between the probes was a factor of three at a relatively strong turbulence intensity, *ε*_S_, of approximately 10^–7.5^ W kg^−1^, while it was within a factor of two at a relatively weak turbulence intensity, *ε*_S_, of approximately 10^–9.5^ W kg^−1^. The dependence of the scatter on the turbulence intensity, *ε*_S_, could be attributed to the shift in the spectra to a higher frequency range, where attenuation is more considerable.

We estimated the time constant, *τ*, for individual thermistors by modifying *ε*_T_ to *ε*_S_ (Fig. S3c,d). The optimal *τ* range was between 3.0 and 10.2 ms for the SP and 1.9 and 5.6 ms for the DP functions. These values represent the ranges of the *τ* uncertainty of the thermistors with unknown time constants and are consistent with the nominal value of the time constant, 7 ± 3 ms for the SP function. Therefore, it was expected that the errors derived from the uncertainty of the time constants would be at least 3.0–10.2 (ms) (SP) or 1.9–5.6 (ms) (DP). The uncertainties of *ε* from the CTD-attached thermistors are discussed based on this result.

### *ε* in the ocean general circulation model

The turbulent energy dissipation data used in the OGCM, which were referred to as TideNF^2^, were compared with the observational data obtained in this study. The model turbulent energy dissipation originally consisted of two types of horizontally 2-D (depth-integrated) data. First, is the energy conversion rate from barotropic to baroclinic (internal) tides (*Ec*(*x*,*y*)), representing the local generation of internal tides, and second, is the local energy dissipation of the internal waves, (*Ed*(*x*,*y*)). Both datasets were obtained from a 3-D high-resolution (1/15°) model forced by tides^3^ and used after being multiplied by 1.5, given that the global baroclinic conversion rate at the limit of zero grid spacing was approximately 1.5 times larger than the grid spacing of 1/15°^2^. A 3-D distribution of energy dissipation rates *ε*_TideNF_ was constructed using three components: (1) the near-field component, *ε*_NEAR_, which represents the local dissipation close to the generation site of the tide-generated internal waves over the rough topography, (2) the far-field component, *ε*_FAR,_ which represents the dissipation of propagating internal waves away from the generation site, and (3) the background component, *ε*_BACK_, which represents the dissipation other than (1) and (2):

1 $$\varepsilon_{{{\text{TideNF}}}} = \varepsilon_{{{\text{NEAR}}}} + \varepsilon_{{{\text{FAR}}}} + \varepsilon_{{{\text{BACK}}}} .$$

The dissipations of the three components were expressed as:

2 $$\begin{aligned} & \varepsilon_{{{\text{NEAR}}}} = E_{{{\text{NEAR}}}} \left( {x,y} \right)F_{{{\text{NEAR}}}} (z)/\rho , \\ & \varepsilon_{{{\text{FAR}}}} = E_{{{\text{FAR}}}} \left( {x,y} \right)F_{{{\text{FAR}}}} (z)/\rho ,\;{\text{and}} \\ & \varepsilon_{{{\text{BACK}}}} = K_{{{\text{BACK}}}} N^{2} /\Gamma , \\ \end{aligned}$$

where *ρ* represents the sea water density (kg m^−3^), *N* represents the buoyancy frequency (s^−1^), *K*_BACK_ represents the background diapycnal diffusivity, and Γ represents the mixing efficiency. *K*_BACK_ = 10^–5^ m^2^ s^−1^ and Γ = 0.2 in the TideNF model^2^. In this study, *N* was computed using the observed density data corresponding to each CTD cast.

*F*(*z*) represents the vertical structures of the near- and far-field components as in a previous study^2^:

3 $$\begin{aligned} & F_{{{\text{NEAR}}}} \left( z \right) = \frac{{e^{{ - \left( {H - z} \right)/h}} }}{{h\left( {1 - e^{ - H/h} } \right)}},\;{\text{and}} \\ & F_{{{\text{FAR}}}} \left( z \right) = 1/H, \\ \end{aligned}$$

where *z* is the depth (m), *H* is the bottom depth (m), and *h* is the decay scale from the bottom (m) of the near-field dissipation. This exponential decay from the bottom topography of the near-field dissipation was based on a previous modelling^25^ and observational study^24^. The far-field component was not based on the observations, and for simplicity, it was assumed to be vertically uniform^2^.

The horizontally variables, *E*_NEAR_ and *E*_FAR_ represent the depth-integrated energy dissipation in the near- and far-field, respectively. They were derived as follows:

4 $$\begin{aligned} & E_{{{\text{NEAR}}}} \left( {x,y} \right) = qEc(x,y)\quad {\text{if}}\quad qEc \le Ed, \\ & E_{{{\text{NEAR}}}} \left( {x,y} \right) = Ed(x,y)\quad {\text{if}}\quad qEc > Ed,{\text{ and}} \\ & E_{{{\text{FAR}}}} \left( {x,y} \right) = Ed(x,y) - E_{NEAR} \left( {x,y} \right), \\ \end{aligned}$$

where *Ec* represents the energy conversion rate from the barotropic tide to the baroclinic internal tide, *Ed* represents the depth-integrated energy dissipation in each water column (the sum of *E*_NEAR_ and *E*_FAR_), and *q* represents the ratio of local dissipation to the generated baroclinic energy (it was set to the constant value, *q* = 0.33)^2,24^. *Ec* and *Ed* were calculated numerically^3^ using 3-D Navier–Stokes equations under hydrostatic and Boussinesq approximations as follows:

5 $$\begin{aligned} & Ec(x,y) = \int\limits_{0}^{H} {\overline{{g\rho^{\prime}w_{s} }} \;{\text{dz}}} ,{\text{ and}} \\ & Ed(x,y) = Ec(x,y) - \int\limits_{0}^{H} {\left[ {\frac{\partial }{{\partial {\it{\text{x}}}}}\left( {\overline{{p^{\prime}u^{\prime}}} } \right) + \frac{\partial }{{\partial {\it{\text{y}}}}}\left( {\overline{{p^{\prime}v^{\prime}}} } \right)} \right]\;{\text{d}}z} , \\ \end{aligned}$$

where *g* represents acceleration due to gravity, $\rho^{\prime}$ represents the deviation of sea water density from the basic field associated with the baroclinic tide motions, *w*_*s*_ represents the vertical velocity resulting from the interaction between the barotropic tidal flow and the bottom topography, and the overbar denotes the time average. *u*′, *v*′, and *p*′ represent the eastward and northward velocities and the pressure perturbations associated with baroclinic tidal motions, respectively.

In this study, the distribution of the energy dissipation rate was examined given that diapycnal diffusivity depends on buoyancy frequency, mixing efficiency, and energy dissipation rate, and these three factors can be different in the model and in the observations. Examples of the spatial distribution of *ε*_TideNF_ using the observed buoyancy frequency field are shown in Fig. 5. *ε*_NEAR_ was large close to the rough bottom topography, which is characterised by a large baroclinic energy. Farther from the bottom, *ε*_TideNF_ was dominated by *ε*_FAR_ and *ε*_BACK_. Additionally, *ε*_FAR_ was assumed to be vertically uniform, and it depends only on the horizontally variable dissipation of remotely generated internal waves. *ε*_BACK_ was large in the upper ocean because it is proportional to *N*^2^. Accordingly, *ε*_BACK_ accounted for more than 20% of all the dissipation rates in the upper 1000-m level.

## Supplementary Information

Supplementary Information.

## Data Availability

The datasets generated during this study are available in the following repository: https://ocg.aori.u-tokyo.ac.jp/omix/GOTO_etal_SREP2020/. MATLAB was used in generating all the figures, except for Fig. 1.

## References

[1] Munk WH (1966). Abyssal recipes. Deep Sea Res. Oceanogr. Abstr..

[2] Oka A, Niwa Y (2013). Pacific deep circulation and ventilation controlled by tidal mixing away from the sea bottom. Nat. Commun..

[3] Niwa Y, Hibiya T (2011). Estimation of baroclinic tide energy available for deep ocean mixing based on three-dimensional global numerical simulations. J. Oceanogr..

[4] Kunze E, Firing E, Hummon JM, Chereskin TK, Thurnherr AM (2006). Global abyssal mixing inferred from lowered ADCP shear and CTD strain profiles. J. Phys. Oceanogr..

[5] Kunze E (2017). Internal-wave-driven mixing: Global geography and budgets. J. Phys. Oceanogr..

[6] Henyey FS, Wright J, Flatté SM (1986). Energy and action flow through the internal wave field: An eikonal approach. J. Geophys. Res..

[7] Gregg MC (1989). Scaling turbulent dissipation in the thermocline. J. Geophys. Res..

[8] Wijesekera H (1993). The application of internal-wave dissipation models to a region of strong mixing. J. Phys. Oceanogr..

[9] Polzin KL, Toole JM, Schmitt RW (1995). Finescale parameterizations of turbulent dissipation. J. Phys. Oceanogr..

[10] Ijichi T, Hibiya T (2015). Frequency-based correction of finescale parameterization of turbulent dissipation in the deep ocean. J. Atmos. Ocean. Technol..

[11] Whalen CB, Talley LD, MacKinnon JA (2012). Spatial and temporal variability of global ocean mixing inferred from Argo profiles. Geophys. Res. Lett..

[12] Whalen CB, MacKinnon JA, Talley LD, Waterhouse AF (2015). Estimating the mean diapycnal mixing using a finescale strain parameterization. J. Phys. Oceanogr..

[13] Waterhouse AF (2014). Global patterns of diapycnal mixing from measurements of the turbulent dissipation rate. J. Phys. Oceanogr..

[14] Garrett C, Munk W (1975). Space-time scales of internal waves: A progress report. J. Geophys. Res..

[15] Polzin KL, Toole JM, Ledwell JR, Schmitt RW (1997). Spatial variability of turbulent mixing in the abyssal ocean. Science.

[16] Klymak JM (2006). An estimate of tidal energy lost to turbulence at the Hawaiian Ridge. J. Phys. Oceanogr..

[17] Nagasawa M, Hibiya T, Yokota K, Tanaka Y, Takagi S (2007). Microstructure measurements in the mid-depth waters of the North Pacific. Geophys. Res. Lett..

[18] Yagi M, Yasuda I (2012). Deep intense vertical mixing in the Bussol'Strait. Geophys. Res. Lett..

[19] Yagi M, Yasuda I (2013). A modified method for estimating vertical profiles of turbulent dissipation rate using density inversions in the Kuril Straits. J. Oceanogr..

[20] Goto Y, Yasuda I, Nagasawa M (2016). Turbulence estimation using fast-response thermistors attached to a free-fall vertical microstructure profiler. J. Atmos. Ocean. Technol..

[21] Goto Y, Yasuda I, Nagasawa M (2018). Comparison of turbulence intensity from CTD-attached and free-fall microstructure profilers. J. Atmos. Ocean. Technol..

[22] Yasuda, I. *et al.* Estimate of turbulent energy dissipation rate using free-fall and CTD-attached fast-response thermistors in weak ocean turbulence. *J. Oceanogr.*10.1007/s10872-020-00574-2 (2020).

[23] Smith WHF, Sandwell DT (1997). Global sea floor topography from satellite altimetry and ship depth soundings. Science.

[24] St. Laurent LC, Toole JM, Schmitt RW (2001). Buoyancy forcing by turbulence above rough topography in the abyssal Brazil Basin. J. Phys. Oceanogr..

[25] St. Laurent LC, Simmons HL, Jayne SR (2002). Estimating tidally driven mixing in the deep ocean. Geophys. Res. Lett..

[26] Melet A, Legg S, Hallberg R (2016). Climatic impacts of parameterized local and remote tidal mixing. J. Clim..

[27] Hibiya T, Ijichi T, Robertson R (2017). The impacts of ocean bottom roughness and tidal flow amplitude on abyssal mixing. J. Geophys. Res. Oceans.

[28] Tanaka Y, Hibiya T, Niwa Y, Iwamae N (2010). Numerical study of K1 internal tides in the Kuril straits. J. Geophys. Res..

[29] Tanaka Y, Yasuda I, Hasumi H, Tatebe H, Osafune S (2012). Effects of the 18.6-yr modulation of tidal mixing on the North Pacific bidecadal climate variability in a coupled climate model. J. Clim..

[30] Hasumi H, Yasuda I, Tatebe H, Kimoto M (2008). Pacific bidecadal climate variability regulated by tidal mixing around the Kuril Islands. Geophys. Res. Lett..

[31] D'Asaro EA (1985). The energy flux from the wind to near-inertial motions in the surface mixed layer. J. Phys. Oceanogr..

[32] Nikurashin M, Ferrari R (2011). Global energy conversion rate from geostrophic flows into internal lee waves in the deep ocean. Geophys. Res. Lett..

[33] Batchelor GK (1959). Small-scale variation of convected quantities like temperature in turbulent fluid Part 1. General discussion and the case of small conductivity. J. Fluid Mech..

[34] Kraichnan RH (1968). Small-scale structure of a scalar field convected by turbulence. Phys. Fluids.

[35] Ruddick B, Anis A, Thompson K (2000). Maximum likelihood spectral fitting: The Batchelor spectrum. J. Atmos. Oceanic Technol..

[36] Gregg MC, Meagher TB (1980). The dynamic response of glass rod thermistors. J. Geophys. Res..

[37] Hill KD (1987). Observations on the velocity scaling of thermistor dynamic response functions. Rev. Sci. Instrum..

[38] Bogucki D, Domaradzki JA, Yeung PK (1997). Direct numerical simulations of passive scalars with Pr > 1 advected by turbulent flow. J. Fluid Mech..

[39] Bogucki DJ, Luo H, Domaradzki JA (2012). Experimental evidence of the Kraichnan scalar spectrum at high Reynolds numbers. J. Phys. Oceanogr..

[40] Peterson AK, Fer I (2014). Dissipation measurements using temperature microstructure from an underwater glider. Meth. Oceanogr..

[41] Lueck RG, Hertzman O, Osborn TR (1977). The spectral response of thermistors. Deep Sea Res..

[42] Gregg MC (1999). Uncertainties and limitations in measuring *ε* and *χ*_T_. J. Atmos. Oceanic Technol..

