# Supplementary material for: Estimation of Basin-scale turbulence distribution in the North Pacific Ocean using CTD-attached thermistor measurements
Source: Sci Rep. 2021 Jan 13;11:969. doi: 10.1038/s41598-020-80029-2 (PMC7807080; doi:10.1038/s41598-020-80029-2)
Supplement: Supplementary file 1 — Supplementary Information. [file 41598_2020_80029_MOESM1_ESM.pdf]

Supplementary Information

Estimation of Basin-scale turbulence distribution in the North Pacific Ocean using CTD-attached thermistor measurements

Yasutaka Goto*[1)4)], Ichiro Yasuda[1)], Maki Nagasawa[1)], Shinya Kouketsu[2)], and Toshiya Nakano[3)5)]

1) *Atmosphere and Ocean Research Institute, The University of Tokyo, Kashiwanoha 5-1-5, Kashiwa, Chiba 277-8564, Japan*

2) *Research Institute for Global Change, Japan Agency for Marine-Earth Science and Technology, Natsushima 2-15, Yokosuka, Kanagawa 237-0061, Japan*

3) *Japan Meteorological Agency, Otemachi 1-3-4, Chiyoda, Tokyo 100-8122, Japan*

Present affiliation:

4）*Kushiro Local Meteorological Office, Japan Meteorological Agency, Saiwai-cho 10-3, Kushiro, Hokkaido 085-8586, Japan*

5）*Nagasaki Local Meteorological Office, Japan Meteorological Agency, Minamiyamate-machi 11-51, Nagasaki, Nagasaki 850-0931, Japan*

*) Corresponding author

Dr. Yasutaka Goto　email: gotoyasutaka@met.kishou.go.jp

Table S1| Cruise and station information. List of cruise name, area, period and number of stations of CTD-attached fast-thermistor measurements. MR denotes the R/V Mirai, KH the R/V Hakuho Maru, RF the R/V Ryofu Maru, and KS the R/V Keifu Maru. Station locations are depicted in Fig. 1.

Cruise name	Area	Period	Number of stations
MR-14-04 (red)	47°N	7/17/2014–8/29/2014	103
KH-16-3 (black)	37.5°N, 41°N	5/31/2016–6/29/2016	40
RF16-06 (light blue)	137°E	7/3/2016–8/24/2016	91
RF16-04 (purple)	East of Japan	4/27/2016–6/1/2016	56
KS16-09 (yellow)	24°N	11/16/2016–12/19/2016	30
KS16-08 (green)	24°N	9/17/2016–10/7/2016	35
KS16-07 (blue)	165°E	7/30/2016–9/12/2016	42
KS16-06 (magenta)	24°N	6/18/2016–7/23/2016	41

Table S2| List of the correlation coefficient in logarithmic scale. Correlation coefficients (r), 95 % confidence intervals of r, and sample sizes. All correlation coefficients are calculated in logarithmic scale.

	correlation coefficient	95% confidence interval	sample size
(Fig. 3a) ε vs N^2	0.74	0.73-0.74	13990
(Fig. 3b) $\int \rho\varepsilon dz$ vs Ec (Ed)	0.58 (0.66)	0.45-0.70 (0.54-0.75)	110 (110)
(Fig. 4a) ε from fine- vs micro-scale	0.64	0.60-0.68	1032
(Fig. 4b) ε from fine- vs micro-scale	0.75	0.74-0.76	5148
(Fig. 8) ε in (a) vs (b)	0.55	0.54-0.56	11368
(Fig. 8) ε in (a) vs (c)	0.50	0.49-0.52	11368

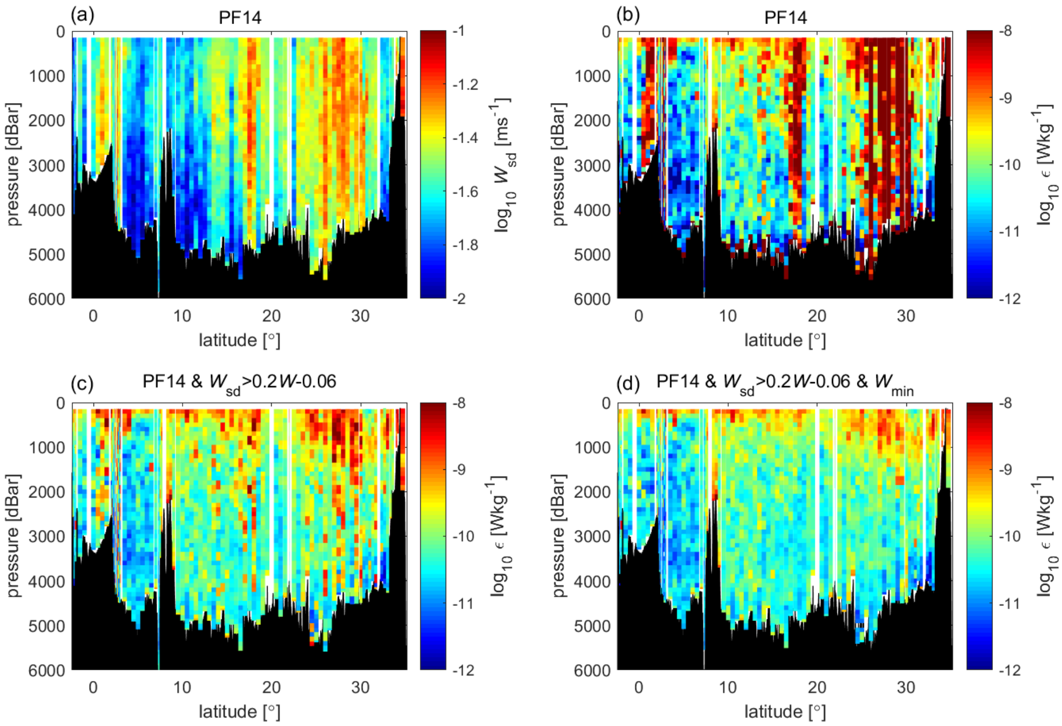

Figure S1| Impact of data screenings on 137°E ε- distribution. Meridional vertical cross-sections of (a) the fall rate (W in ms^{-1}) variability represented by the standard deviation W_{sd} and (b–d) the arithmetically averaged kinetic energy dissipation rate ε in each 200 m depth range. (b) ε applying the data rejection used in Peterson and Fer (2014) (PF14); mean absolute deviation, signal and noise ratio, and likelihood ratio[35,40]. (c) ε after the data screening with the rejection criteria of $W_{sd} > 0.2W–0.06$, and (d) ε after the further rejection of data at the minimum of the fall rate W_{min}.

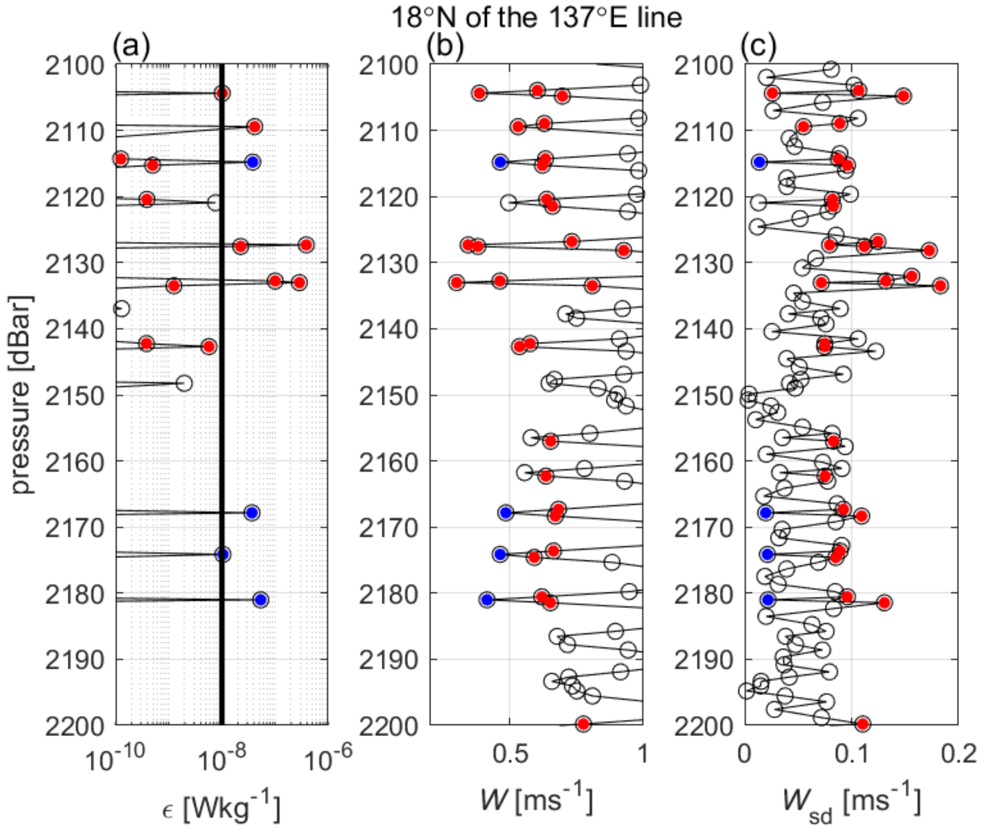

Figure S2| Abnormal data near the fall-rate minimum. An example of the vertical profiles of (a) ε, (b) W, (c) W_{sd} for 1 s bin at 18°N along the 137°E section, obtained during the RF16-06 cruise. The circles are data, which passed the criterion[40] tests. The red dots satisfy $W_{sd} > 0.2W–0.06$. The blue dots satisfy $W_{sd} < 0.2W–0.06$, $\varepsilon > 10^{-8}$, and W_{min} (local minimum of W).

Table S3| List of FP07 probes in Fig. S3. Serial numbers (S/N) of FP07 probes, cruise and ship, period (year/month), and area of VMP2000 observations. T1 and T2 indicate the first and second probes.

S/N of FP07	Cruise name	Ship name	Term	Area	Number of samples
271 (T1)	KH-09-4	Hakuho-maru	Aug.–Sep. 2009	Aleutian	1292
285 (T2)	KH-09-4	Hakuho-maru	Aug.–Sep. 2009	Aleutian	1281
415 (T1)	Go11	Gordienko	Jul.–Aug. 2011	Kuril	4626
883 (T1)	Mu14	Multanovskiy	Jun.–Jul. 2014	Kuril	761
886 (T2)	Mu14	Multanovskiy	Jun.–Jul. 2014	Kuril	765
1024 (T1)	KH-16-3	Hakuho-maru	Jun. 2016	NW Pacific	3649
1025 (T2)	KH-16-3	Hakuho-maru	Jun. 2016	NW Pacific	3622

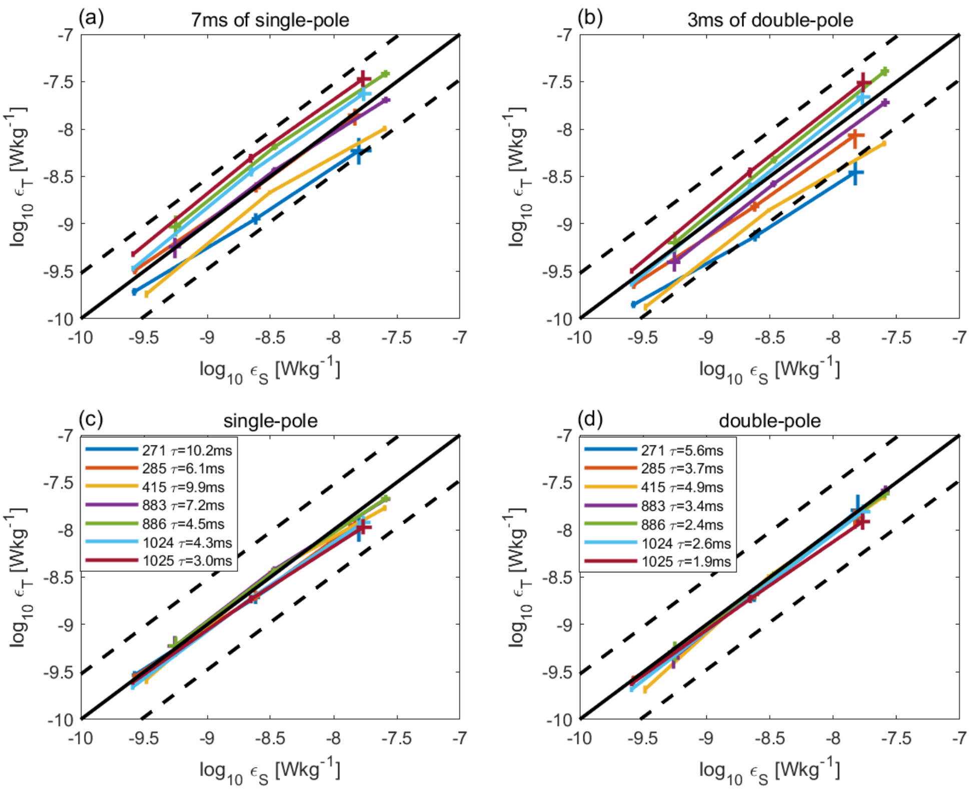

Figure S3| ε estimate uncertainties in individual FP07 probes. Dependence of the ε_T - ε_S relationship on the time constant τ of individual FP07 thermistors. The plus marks with lines are the geometric means with 95% confidence intervals of the bootstrap method of ε_S and ε_T in the ranges of $10^{-(i+1)} < \varepsilon_S < 10^{-i}$ (i = 7, 8, 9). (a) single-pole correction with τ = 7 ms and (b) double-pole correction with τ = 3 ms. (c, d) For cases where optimally computed τ by minimizing $\log_{10}|\varepsilon_T/\varepsilon_S|$ are used for (c) single- and (d) double-pole corrections.
